# Housing in a Large Open Cage Did Not Affect the Phenotypic Traits of Obese Male Zucker fa/fa Rats When Compared to IVC-Housed Rats, but Improved the Rats’ Well-Being

**DOI:** 10.3390/ani15182687

**Published:** 2025-09-13

**Authors:** Oddrun Anita Gudbrandsen

**Affiliations:** Dietary Protein Research Group, Centre for Nutrition, Department of Clinical Medicine, University of Bergen, 5021 Bergen, Norway; oddrun.gudbrandsen@uib.no

**Keywords:** 3Rs, animal welfare, animal well-being, obese phenotype, housing condition, laboratory animal science, environmental enrichment

## Abstract

The housing of laboratory rats in cages with dimensions according to international standards for research animals can hardly be regarded as a stimulating environment, even when fulfilling the minimum requirements for environmental enrichment. Little is known about whether changes in the housing situations to improve living conditions will affect the phenotypic traits of well-known models for human diseases. The obese Zucker fa/fa rat develops hyperlipidemia, hypertension, and fatty liver, and is widely used for studies on metabolic complications of obesity in humans. Young male obese Zucker fa/fa rats were housed in pairs in standard individually ventilated cages (IVCs: floor area 1500 cm^2^ and maximum height 20 cm), or 4–6 rats were housed in a large open cage (LOC: floor area 7705 cm^2^ and height 75 cm). The LOC provided an environment with more physical, social, auditory, visual, olfactory, and tactile stimuli compared to IVCs. The aims were to compare the development of obesity comorbidities and to assess the well-being of rats housed under different conditions. The rats housed in IVCs and the LOC had similar adiposity, blood pressure, hepatic triacylglycerol content, and similar serum concentrations of cholesterol, triacylglycerol, and alanine transaminase. IVC-housed rats showed some signs of distress, such as less interest in nest-building and signs of apathy compared to LOC-housed rats. To conclude, LOC housing did not affect the typical phenotype of obese Zucker fa/fa rats but did improve the welfare of these rats.

## Simple Summary

Laboratory rats are commonly housed in cages with limited physical, social, auditory, visual, olfactory, and tactile stimuli. Better housing conditions can improve the lives of research animals and will most likely generate better research results that are more transferable to humans and result in more reproducible experiments when animal discomfort is reduced. This study investigated the development of obesity-related disturbances in a rat model frequently used for diseases accompanying obesity in humans. The rats were either housed in standard individually ventilated cages (IVCs) or in a large open cage (LOC). The LOC provides more space and possibilities for environmental enrichment, as well as allowing more opportunities for rats to adhere to their natural behavior in an environment that stimulates them socially, cognitively, and physically. The developments of high levels of blood lipids (including cholesterol), high blood pressure, and fatty liver were similar in rats housed in IVCs or an LOC, whereas the well-being of the rats was improved by LOC housing. This means that IVCs can be replaced by LOCs without affecting the typical traits for this particular rat model.

## 1. Introduction

The housing conditions of small laboratory animals, such as the rat, are regulated through guidelines specifying the minimum floor area required per animal when being housed in groups, with recommendations provided for environmental enrichment for experiments conducted in the European Union [1], Norway [2], the United Kingdom [3], and the United States of America [4]. Within this limited space, there is little room for environmental enrichment to allow for natural behavioral opportunities such as foraging, sheltering, exploring, nest building, and gnawing. Baumans and Van Loo [5] define environmental enrichment as any modification in the animals’ environment with the purpose of improving physical and psychological well-being through stimuli that meet their species-specific needs, with focus on social environment (social partners, including humans) and physical environment (sensory stimuli and nutritional aspects). Social housing, nesting materials, shelter, forage opportunities, and larger cages were proposed as basic housing components for research rats in a recent metareview by Ratuski and Weary [6].

The obese Zucker fa/fa rats become obese before the age of 5 weeks, and spontaneously develop hyperlipidemia, hypertension, and fatty liver whilst being normoglycemic [7,8]. These rats developed obesity due to a defect in the leptin receptor [9], resulting in hyperphagia [7]; in addition, they have higher efficiency for food utilization, a higher rate of lipogenesis in adipose tissues and in the liver [7,10], and lower capacity for fatty acid oxidation in the liver [10] compared to lean rats. The obese Zucker fa/fa rats can be perceived as docile, and their rapid weight gain due to efficient fat storage may be a limiting factor for their capability and willingness to be physically active; however, based on personal experience, they are curious and eager to investigate the world outside their cage when given the opportunity. The obese Zucker fa/fa rat is a much-used model for studies on metabolic complications and possible treatments of obesity, since they present a range of abnormalities similar to those seen in humans with obesity [11], and concern may be raised with regard to whether housing in larger cages with ample opportunities for physical activity will affect the phenotype of this strain. The aims of the present study were therefore to investigate if the phenotypic traits of male obese Zucker fa/fa rats were affected when housed in a large open cage (LOC) compared to housing in standard individually ventilated cages (IVCs), and to document the well-being of the rats through physiological and behavioral assessments. In accordance with the 3Rs (Replacement, Reduction, and Refinement) [12], historical data from obese Zucker fa/fa rats housed in IVC were used for a comparison against housing in an LOC to minimize the number of research animals used. The hypothesis for this study was that housing obese Zucker fa/fa rats in an LOC would not affect the typical phenotype of their rat strain when compared to housing in IVCs, based on the strain’s known characteristics of hyperphagia and disturbances in lipid metabolism. Standard laboratory cages interfere with natural behavior such as burrowing, climbing, standing, and stretching upright [13,14]. It was therefore expected that LOC housing would result in better animal welfare. The present findings suggest that LOC housing did not affect the phenotype of obese Zucker fa/fa rats but improved the rats’ well-being.

## 2. Materials and Methods

### 2.1. Ethical Statement

The study protocols for the included experiments were approved by the National Animal Research Authority (Norway) in accordance with the Animal Welfare Act and the Regulation of animal experiments (Approval No. 11603 and 29717). All applicable guidelines for the care and use of animals were followed.

### 2.2. Design

Data from three experiments are included in this study, all using male obese Zucker fa/fa rats (HsdHlr:ZUCKER-Leprfa) from Harlan Laboratories (Indianapolis, IN, USA). All rats had ad libitum access to feed and water. The rats used in the present study represent the control groups in the three experiments. In all three experiments, the rats were randomly allocated to the control group or the intervention diet groups by drawing paper lots from a jar, and the carer was blinded to group allocation since rats and diets were coded by numbers that were not identifiable. Data are presented only for rats allocated to the control group in these three experiments. Rats were weighed weekly. The rats in all three experiments were housed in the same animal room and were fed the same standard diet. Since the diet was in the form of powders, the rats’ incisors were checked for malocclusion every week to ensure that their teeth did not overgrow. The rats in all three experiments were handled by the same carer. The rats were housed in a room with a controlled light/dark cycle (dark 20:00–06:00). Rats were housed individually in metabolic cages (Ancare Corp., New York, NY, USA) for one period of 24 h for the measurement of feed intake. Rats were euthanized in a fasting state (6–12 h), whilst anesthetized with isoflurane (Isoba vet, Intervet, Schering-Plough Animal Health, Boxmeer, The Netherlands). Blood was drawn from the heart and collected in BD Vacutainer SST II Advance gel tubes (Becton, Dickinson and Company, Franklin Lakes, NJ, USA) for isolation of serum. Liver and epididymal white adipose tissue (WATepi) were removed. Serum and organs were frozen at −80 °C.

#### 2.2.1. Experiments 1 and 2 (IVC)

The first two studies were conducted in 2019 (Experiment 1: N = 6, age 8 weeks at intervention start, and age 12 weeks at euthanasia) and in 2022 (Experiment 2: N = 6, age 8 weeks at intervention start, and age 14 weeks at euthanasia). The rats were housed in pairs in individually ventilated cages (IVC type 4, blue line, Tecniplast, Buguggiate, Italy), under standard conditions with temperatures of 23–25 °C and 22–23 °C, respectively. The IVC-4 has a floor area of 1500 cm^2^, a maximum height of 20 cm, and was equipped with one gnawing block (Aspen brick, 100 × 20 × 20 mm, TAPVEI^®^ Harjumaa, Estonia), three paper sachets containing soft wood bedding for nesting material (2HK Nestpak, Datesand Ltd., Manchester, UK), and one red polycarbonate hut (Fat Rat Hut, 150 × 165 × 85 mm, Datesand, Stockport, UK). The rats were fed standard chow (V1536, ssniff Spezialdiäten GmbH, Soest, Germany) until they were 8 weeks old, followed by a gradual shift over 4 days to a semi-purified standard powder diet (AIN-93G) for growing rodents [15]. Body weight gain, the relative weight of WATepi, mean arterial blood pressure (MAP), and serum alanine transaminase (ALT) concentration from Experiment 1 were published [16].

#### 2.2.2. Experiment 3 (LOC)

**Figure 1:**
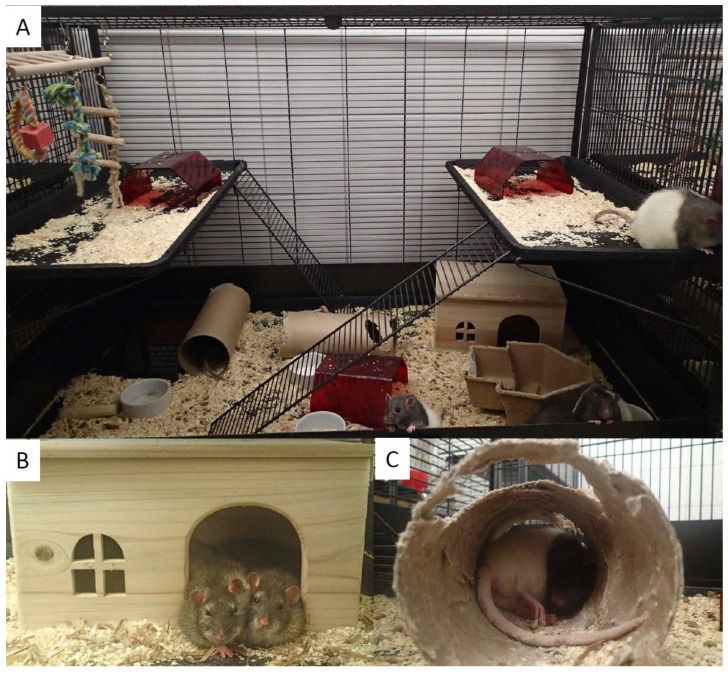
The upper half of the LOC housed six rats (**A**), with two rats in the wooden house (**B**) and one rat relaxing in a cardboard tunnel (**C**).

The third experiment, conducted in 2023, comprised 10 rats aged 12 weeks at euthanasia. The rats were housed in pairs in IVC-4 with free access to chow (Teklad Global Diet 2018 S, Inotiv, West Lafayette, IN, USA) until they reached 7 weeks of age. Rats were gradually habituated to a semi-purified standard powder diet (AIN-93G) for growing rodents [15] for 4 days before they were moved to the LOC, with one short familiarization visit (approximately 15 min) in the large cage on each of the last two days in the previous cage. The LOC had a floor area of 7705 cm^2^ and a height of 150 cm (Suite Royale XL, Savic^®^, Kortrijk, Belgium) and consisted of two plastic floors and four plastic platforms with metal ladders. The cage was split into two equal-sized halves, each 75 cm high, with 2HK Nestpak on plastic floors and platforms, housing six rats on the upper half and four rats on the lower half of the cage. Both halves of the LOC were equipped with the following environmental enrichments: three Fat Rat Huts (red polycarbonate, Datesand, Stockport, UK), two cardboard tunnels (Play Tunnel, 100 × 50.8 × 1.25 mm, Datesand), two gnawing blocks (Aspen brick, 100 × 20 × 20 mm, TAPVEI^®^), one Rat Corner Home (paper pulp, 23 × 19 × 9.5 cm, Datesand), two 8g portions of Bed-R’Nest nesting material (kraft paper, The Andersons, Inc., Maumee, OH, USA), one wooden house (pine, 28 × 16 × 18 cm, Trixie Heimtierbedarf GmbH & Co, Tarp, Germany), one wooden playing roll with a bell (Trixie), one suspension bridge consisting of a wooden ladder and rope ladder, play rope and rope ring with a wooden block (Trixie), one or two plastic bottles with water (Classic Crystal Deluxe Water Drinking Bottle, Caldex Holdings Ltd., Halifax, UK), and ceramic bowls with water or powder feed (Figure 1). The room temperature was 22 °C.

### 2.3. Assessment of Animal Welfare

The guideline from The European Commission Working document on a severity assessment framework includes six high-level categories for evaluation of laboratory animal welfare; these include appearance, body functions, environment, behaviors, procedure-specific indicators, and free observations for observers [17]. Table 1 presents the criteria for evaluation of animal welfare in Experiments 1–3, which were adapted from nutritional studies on the obese Zucker fa/fa rat based on more than 25 years of personal experience using this model. The same carer handled and assessed all rats in the three experiments. The rats were observed at the same time of day (early in the morning, shortly after the light was turned on at 6 a.m.) and for the same duration of time (>1 h) in all three experiments.

### 2.4. Analyses

The caloric content of the AIN-93G diet was determined using a bomb calorimeter method in accordance with ISO 9831:1998 [18] and a Parr 6400 calorimeter (Parr Instrument Company, Illinois, IL, USA).

Body weight was recorded weekly in all three experiments. Adiposity was estimated based on the weight of WATepi.

Blood pressure was measured in conscious rats in Experiments 1 and 3 using the tail-cuff method (CODA-6, Kent Scientific Corporation, Torrington, CT, USA). The rats were placed in a heating cabinet at 32 °C for 30 min before blood pressure was measured. The mean arterial pressure (MAP) was calculated as follows: diastolic blood pressure + 1/3 [systolic blood pressure—diastolic blood pressure]. The same operator performed all blood pressure measurements in both experiments. The rats were hand-tamed and trained to be in the constrainer before the measurements. The rats were placed in holders on a warming platform (both from Kent Scientific Corporation). Ten cycles with a 5 s delay between cycles (without acclimatization cycles in advance) were measured under close monitoring by the operator. Max occlusion pressure was 250 mmHg, deflation time was 15 s, and the minimum volume was 15 µL.

Serum triacylglycerols, total cholesterol, and alanine aminotransferase were quantified on the Cobas c111 system (Roche Diagnostics, Roche Diagnostics GmbH, Mannheim, Germany) using the TRIGL (Triglycerides), CHOL2 (cholesterol Gen.2), and ALTL (alanine aminotransferase acc. IFCC, measured with pyridoxal phosphate activation) kits from Roche Diagnostics.

Lipids were extracted from the liver using a mixture of methanol and chloroform, as described by Bligh and Dyer [19]. The lipid extracts were evaporated to dryness under nitrogen and re-dissolved in isopropanol before quantification of triacylglycerols on the Cobas c111 system using the TRIGL (Triglycerides) kit from Roche Diagnostics.

### 2.5. Outcome Measurements

The outcomes were to investigate the effect of different housing conditions (IVCs versus an LOC) on the phenotypic traits of male obese Zucker fa/fa rats, specifically adiposity, hyperlipidemia, high blood pressure, and fatty liver, as well as assessments of physical and behavioral indicators of animal welfare according to the guidelines from The European Commission Working document on a severity assessment framework [17].

### 2.6. Statistical Analyses

The data included in the present study were collected from the control groups in three individual experiments using male obese Zucker fa/fa rats of the same age and from the same breeder, housed in the same animal room, and fed the same diet. In line with the 3Rs (Replacement, Reduction, and Refinement) [12], historical data from housing in IVCs were used for comparison to housing in an LOC to minimize the number of research animals used. Since data on effect size were not available for sample size calculation or minimally detectable effect sizes, the necessary sample size could not be estimated, and this study will contribute to sample size calculations for future studies with similar designs.

Statistical analyses were conducted using SPSS Statistics version 28 (SPSS, Inc., IBM Company, Armonk, NY, USA). All data were evaluated for normality using the Shapiro–Wilk test, revealing that most variables were normally distributed. Serum ALT was not normally distributed, and data were log-transformed before analyses. The body weights for LOC-housed rats (Experiments 1 and 2) were compared with that of IVC-housed rats (Experiment 3) using one-way ANOVA. Student’s *t*-test was used to compare the phenotypic traits between Experiments 1 and 3, where rats were 12 weeks old at euthanasia. The statistical analyses were conducted with N = 6 rats in Experiments 1 and 2 (IVC), and with N = 10 in Experiment 3 (LOC). The cut-off value for statistical significance was set at a probability of 0.05. No statistical analyses were applied to the indicators for welfare.

## 3. Results

### 3.1. Animal Welfare

The male obese Zucker fa/fa rat spontaneously develops obesity at a young age but appears healthy until comorbidities develop. The current experiments were terminated before serious comorbidities were established, as evidenced by the measurements below. The criteria used for assessing the animal welfare in Experiments 1–3 were adapted based on previous experiences from nutritional studies in obese Zucker fa/fa rats of comparable age. None of the rats in either of the three experiments lost weight, showed evidence of a lack of grooming or fecal/urine staining on the fur, had skin lesions, abnormal porphyrin staining, malocclusion, increased or decreased feed/water intake, loose stools or diarrhea, or lethargy > 1 h after blood pressure measurements, and were considered normal (score 0) with regard to these indicators (Table 2). Rats in the IVCs generally showed no or little interest in nest building as no nests were observed in any of the IVCs, whereas rats in the LOC manipulated the environment by moving the bedding materials up and down between the platforms and around on the main floor, built circular paper nests inside or outside the wooden houses, horizontally rearranged the positions of the cardboard tunnels and Rat Corner Homes, and gnawed the cardboard tunnels and the paper pulp Rat Corner Home to pieces. The rats in the LOC alternated between sleeping or relaxing together in the wooden house or in the cardboard tunnels or solitary in the cardboard tunnels, behind the wooden house, in the Fat Rat Hut or in the Rat Corner Home. The rats in the IVC slept anywhere in the cage, either together or separately, and did not use the paper sachets as nesting material. The IVC rats gnawed on the wooden blocks but showed little interest in using enrichment items and were scored 1–3. The LOC rats gnawed on both wooden blocks and most of the other chewable inventory in the cage.

The rats’ behavior was only observed during normal working hours, that is, during the light phase. The LOC rats were active and ran up and down the ladders and engaged in playfighting when awakened by the carer entering the room during the light phase. The IVC rats were rarely observed performing any physical activity during the day. No change from normal temperament was observed between the cage mates in the IVC or in the LOC, which was supported by no observations of bite wounds, and no blood were seen on the rats or in the cages. Also, no aggression towards the carer was observed in rats from either cage type. A certain degree of apathy was observed in rats housed in the IVC, as these rats did not show any interest in greeting the carer and allowed themself to be handled without resistance. The LOC rats were contact-seeking and approached the carer to be petted and showed great interest in human contact by initiating play through careful biting and licking at fingers and hands when handled. No unexpected observation was registered under the free observation category in either of the three experiments.

### 3.2. Measurements

**Figure 2:**
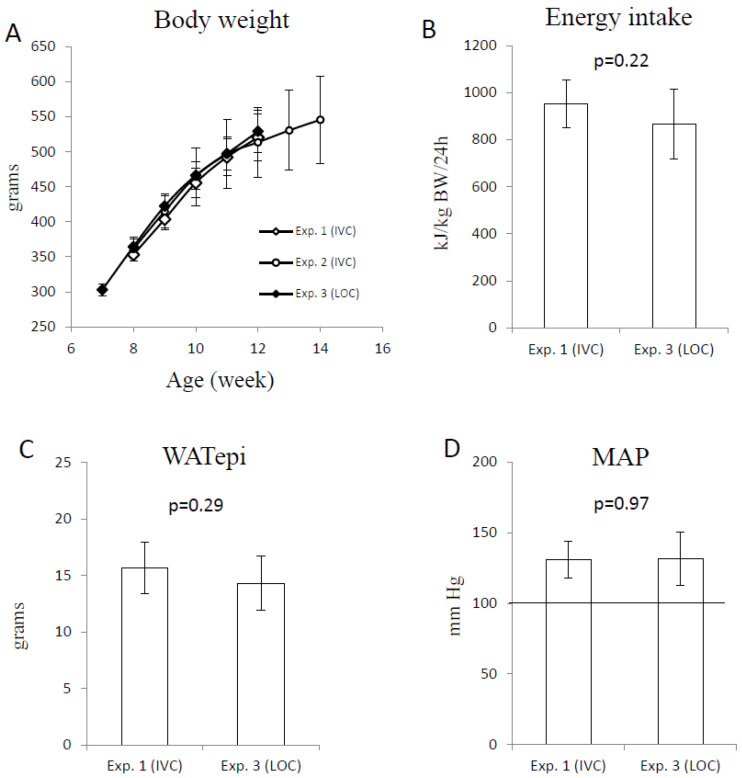
Body weight recorded weekly for Experiments 1 (IVC, N = 6), 2 (IVC, N = 6), and 3 (LOC, N = 10) (**A**), and daily energy intake (**B**), weight of WATepi (**C**), and MAP (**D**) for Experiments 1 and 3. Data are presented as the mean with standard deviation. The group’s bodyweight was compared using one-way ANOVA, and daily energy intake, WATepi weight, and MAP were compared using Student’s *t*-test, with the cut-off value for statistical significance set at a probability of 0.05. No differences were seen between the groups. The horizontal line in (**D**) represents the reference value for MAP in 12-week-old male lean Zucker fa/fa rats [8]. IVC; individually ventilated cages, LOC; large open cage, MAP; mean arterial blood pressure, WATepi; epididymal white adipose tissue.

The typical phenotypic traits of the obese Zucker fa/fa rat were measured to investigate if these were affected by the housing conditions, that is, housing in an LOC vs. IVC. Body weights recorded from 8 to 12 weeks of age (the age-period in common for all three experiments) were similar between the three experiments (Figure 2A). The mean daily energy intake (Figure 2B), the WATepi weight (Figure 2C), and the blood pressure (MAP, Figure 2D) were similar between the two groups of 12-week-old rats in Experiments 1 and 3.

**Figure 3:**
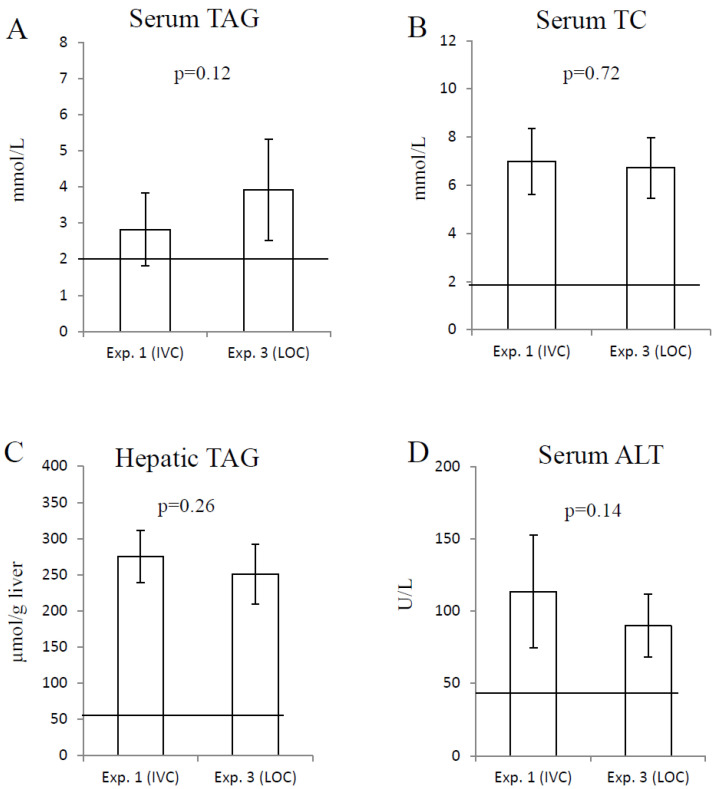
Serum triacylglycerol concentration (**A**), serum total cholesterol concentration (**B**), hepatic triacylglycerol content (**C**), and serum alanine transaminase concentration (**D**) for Experiment 1 (IVC, N = 6) and Experiment 3 (LOC, N = 10). Data are presented as the mean with standard deviation. The horizontal lines represent serum chemistry reference values for healthy male rats < 6 months [20] and the commonly used limit for fatty liver, which is defined as a triacylglycerol content above 5 wt% (corresponding to approximately 60 µmol triacylglycerols/g liver). Groups were compared using Student’s *t*-test, with the cut-off value for statistical significance set at a probability of 0.05. No differences were seen between the groups. ALT; alanine transaminase, IVC; individually ventilated cages, LOC; large open cage, TAG; triacylglycerol, TC; total cholesterol.

The two groups of 12-week-old rats in Experiments 1 and 3, housed in an IVC and LOC, respectively, were also similar with regard to serum triacylglycerol concentration (Figure 3A), serum total cholesterol concentration (Figure 3B), hepatic triacylglycerol content (Figure 3C), and serum ALT concentration (Figure 3D).

## 4. Discussion

The obese Zucker fa/fa rat is a widely used model for human obesity and comorbidities, and it was therefore of interest to investigate whether the phenotype of this strain was affected by housing in a cage that was considerably larger than the standard-sized IVC. Since housing in LOC allows the rats to be more physically active, this study aimed to examine if the development of obesity and comorbidities of obesity, that is, hyperlipidemia, high blood pressure, and fatty liver, were affected by the different housing conditions. Alongside this, physical indicators for well-being were assessed.

In accordance with the 3Rs [12], historical data for IVC-housed obese Zucker fa/fa rats were used in order to reduce the number of research animals in comparison to rats of the same strain housed in LOC. To ensure that the experimental conditions were as similar as possible between the experiments, the rats were the same age, all were males, provided from the same breeder, the rats from all three experiments were housed in the same animal room at our animal facility, they were fed the same standard AIN-93G diet, and they were handled by the same carer. The similar body weight gain from age 8 to 12 weeks in all three experiments, and the similar energy intake, adiposity, and MAP between Experiments 1 and 3, show that these characteristic phenotypic traits for obese Zucker fa/fa rats were not affected by the different housing conditions, as rats housed in either the IVC or LOC comparably developed obesity and high blood pressure. Also, the similar serum concentrations of triacylglycerol, total cholesterol and ALT, as well as the hepatic content of triacylglycerol between Experiments 1 and 3, indicate that the different housing conditions did not alter the development of dyslipidemia and fatty liver in this rat strain. These findings suggest that the IVC can be replaced by LOC without affecting the typical phenotypic traits for obese Zucker fa/fa rats.

The use of larger cages that allow for social housing and more natural behavior is recommended [6] and should be considered by nutrition scientists when conducting studies in healthy rodents that do not require IVCs for protection against pathogens. Unfortunately, many laboratory rats lack sufficient space to allow for natural behavior and are housed in cages with no possibilities for climbing, digging, or foraging, and a recent metareview found that many researchers are concerned that environmental enrichment may affect research outcomes [6]. Research animals housed in conventional cages with little or no environmental enrichment show signs of poor welfare such as abnormal behavior, compromised sleep quality, and increased risk for cognitive depression [21]. This might be no surprise, since chronic stress in humans is associated with increased risk for detrimental effects such as depression, fear, anxiety, and changes in neurohormonal systems as well as immune, endocrine, and cardiovascular systems [22]. Thus, it is likely that nutrition studies using rats and mice may become more relevant for humans if housing conditions were improved.

The well-being of the rats in the present paper was assessed through documenting physiological and behavioral health indicators according to the six high-level categories for the evaluation of laboratory animal welfare recommended in the guidelines from The European Commission Working document on a severity assessment framework [17]. The appearance, body functions, procedure-specific indicators, and free observations of the rats were scored as normal, whether housed in the IVC or LOC, as could be expected since the rats were euthanized before they developed serious comorbidities. The apathy and the lack of interest in nest building in the IVC-housed rats are alarming observations and indicate that these rats were in distress. It is possible that the IVC-housed rats did not find the paper sachets satisfactory for the construction of nests, and that the access to other nesting materials, such as Bed-R’Nest nesting material, would have stimulated nest-building. In contrast, the LOC-housed rats showed no signs of apathy as they were curious and greeted the carer when approaching the cage; they built proper nests and arranged places to relax and hide on different places in the cage. Hopefully, in the future, dedicated scientists will give greater priority to providing housing conditions for research animals that enable the animals with normal physiology and possibilities to perform their natural behaviors, whenever this is conceivable, to ensure good science, in an environment that stimulates the animals both socially, cognitively, and physically [23,24,25,26]. Better housing conditions will improve the life of the research animals and will most likely result in better research results that are more transferable to humans and result in more reproducible experiments when the animals’ discomfort is reduced.

From this author’s perspective, the experience of using an LOC was unambiguously positive, and the estimated time spent per rat housed in the LOC was approximately equal to that of IVC-housed rats. The purchase of the LOC and a variety of disposable and reusable forms of environmental enrichment came at a cost that was considered to be within a reasonable range when considering the improved well-being of the rats. The feedback from the animal facility staff was that they enjoyed watching, hearing, smelling, and interacting with the rats. The rats also enjoyed the visits of the staff, as evidenced by the curiosity the rats displayed by sniffing in the air and seeking the closest corner to the visitors’ position.

The present study has some limitations that should be considered when interpreting the findings, especially the use of historical data for IVC-housed rats compared to rats housed in the LOC. However, efforts were made so that the conditions were as similar as possible, by using only male rats of the same age and from the same breeder, housing in the same animal room, the same standard AIN-93G diet, and the same carer to handled and assessed all rats in the three experiments. The present study is small, and the findings may not be universal for all rat strains, ages, and both sexes, but it shows an important finding regarding the choice of cage when considering animal welfare. Statistical analyses are often a challenge when working with social research animals such as rats, since many of the most used statistical methods including ANOVA and the Independent samples *t*-test for normal-distributed data and the non-parametric tests, such as the Mann–Whitney U test and Kruskal–Wallis test, assume that the observations within each group (the experimental units) are independent of each other. Thus, the assumption of independent observation may be false for rats housed in groups; however, the singular housing of rats should be avoided when possible. One might assume that the impact of a cage-mate will be more pronounced when rats are housed in pairs in a small IVC with a floor area of 1500 cm^2^ compared to the impact in an LOC. In order to reduce the impact of any dominant male on access to feed and hiding places compared to its cage-mates in the LOC used in the present study, the cage was equipped with several feed bowls and options for places to hide and nest.

## 5. Conclusions

LOC housing of obese Zucker fa/fa rats for 5 weeks did not change their characteristic phenotypic traits of obesity, hypertension, hyperlipidemia, and fatty liver compared to those housed in an IVC, even though the LOC allowed for more natural behavioral opportunities and, thus, more possibilities for physical activity compared to the IVC. The rats housed in the IVC showed signs of apathy and had little interest in nest building, which are signs of distress, whereas no adverse effects of LOC housing were observed. These findings suggest that using a LOC with more possibilities for environmental enrichment does not affect the phenotype of the Zucker fa/fa rat model, but has a favorable impact on animal welfare.

## Figures and Tables

**Table 1:** Criteria for the assessment of animal welfare in Experiments 1–3.

High-Level Categories	Areas to Focus on When Observing Rats	Specific Indicators to Monitor	Action/Clinical Scores ^1^
Appearance	Body condition	Weight loss	5–10% weight loss: 1–2 20% weight loss: 4 >20% weight loss: HEP
	Coat and skin condition	Lack of grooming Fecal or urine staining	Feces in fur at the anus (wash with lukewarm water): 1 Recurring episodes with feces in fur >48 h: 3 Recurring episodes with feces in fur >72 h combined with lack of grooming: HEP
		Skin lesions Injury/wound	Palpation of rats at 3/week to check for bite wounds and swellings. Treat/wash wounds if necessary. Small bite wounds: 1 Small bite wounds that do not start to heal after 48 h: 4 Wounds that do not heal or are infected: HEP
	Discharge	Porphyrin staining	Normal amount: 0–1 Increased amount: 2–3
	Mouth	Malocclusion, Broken teeth	Weekly checks of teeth and feed intake; cut teeth before they become too long or cause problems for eating
Body functions	Feed/water intake	Increased/decreased intake	Monitor feed and water intake, and monitor body weight. Change feed if necessary.
Environment	Enclosure environment, including any litter, nesting material, and enrichment items	Presence and consistency of feces	Loose stools or diarrhea: 2 Diarrhea >48 h: 4
		Whether rats are using enrichment items, e.g., nesting material, taking refuge, or using wooden chewing blocks	Nest building and chewing on blocks: 0 Slightly disorganized nesting: 1 Some attempts but unorganized and not chewing on blocks: 3
Behaviors	Social interaction	Change from normal temperament; apprehensive or aggressive interactions with other rats; anxiety Isolated or withdrawn from other animals in social group	Normal behavior: 1 Aggressive: 3 Passive or withdrawn from cage-mates: 5
	Undesirable behaviors	Apathy	Slightly apathic: 1 No interest in greeting carer: 3 Not eating: HEP
		Increased aggression to humans or cage-mates	Tense and nervous on handling: 1 Markedly distressed/aggressive on handling: 3 Can no longer be handled: HEP
Procedure-specific indicators	Restitution after blood pressure measurement	Not recovered 1 h after measurements	Lethargic after 1–2 h: 1–2 Lethargic after >3 h: 4 Lethargic after >6 h and not drinking: HEP
Free observations		A severity assessment scheme was available for registration of any observations of unexpected negative welfare impacts.	

^1^ Actions according to individual scores: Score 0: Normal condition/behavior; Score 1: Review frequency of monitoring; Score 2–3: Consider supplementary care; Score 4–5: Consult veterinarian; Score HEP (6): Implement humane endpoint. Adapted from [17].

**Table 2:** Observation and score sheet for rats housed in the IVC (Experiment 1 and 2) or LOC (Experiment 3).

High-Level Categories	Areas to Focus on When Observing Rats	Specific Indicators to Monitor	Clinical Scores for IVC Rats	Clinical Scores for LOC Rats
Appearance	Body condition	Weight loss	0	0
	Coat and skin condition	Lack of grooming Fecal or urine staining	0	0
		Skin lesions	0	0
	Discharge	Porphyrin staining	0–1	0–1
	Mouth	Malocclusion Broken teeth	0	0
Body functions	Food/water intake	Increased/decreased intake	0	0
Environment	Enclosure environment	Presence and consistency of feces	0	0
		Whether animal is using enrichment items	1–3	0
Behaviors	Social interaction	Change from normal temperament	0	0
	Undesirable behaviors	Apathy	3	0
		Increased aggression to humans or cage-mates	0	0
Procedure-specific indicators	Restitution after blood pressure measurement	Not recovered 1 h after measurements	0	0
Free observations			0	0

Actions according to individual scores: Score 0: Normal condition/behavior; Score 1: Review frequency of monitoring; Score 2–3: Consider supplementary care; Score 4–5: Consult veterinarian; Score HEP (6): Implement humane endpoint. Adapted from [17].

## Data Availability

The raw data supporting the conclusions of this article will be made available by the author on request.

## Abbreviations

The following abbreviations are used in this manuscript:

ALT	Alanine transaminase
IVC	Individually ventilated cages
LOC	Large open cage
MAP	Mean arterial blood pressure
WATepi	Epididymal white adipose tissue
